# Consciousness Detection on Injured Simulated Patients Using Manual and Automatic Classification via Visible and Infrared Imaging

**DOI:** 10.3390/s21248455

**Published:** 2021-12-18

**Authors:** Diana Queirós Pokee, Carina Barbosa Pereira, Lucas Mösch, Andreas Follmann, Michael Czaplik

**Affiliations:** Acute Care Innovation Hub, Department of Anaesthesiology, RWTH Aachen University Hospital, 52074 Aachen, Germany; cbarbosapere@ukaachen.de (C.B.P.); lmoesch@ukaachen.de (L.M.); afollmann@ukaachen.de (A.F.); mczaplik@ukaachen.de (M.C.)

**Keywords:** disaster medicine, triage, mass casualty incidents, remote imaging, infrared imaging, movement detection, image processing, visible imaging, mask R-CNN, UAV

## Abstract

In a disaster scene, triage is a key principle for effectively rescuing injured people according to severity level. One main parameter of the used triage algorithm is the patient’s consciousness. Unmanned aerial vehicles (UAV) have been investigated toward (semi-)automatic triage. In addition to vital parameters, such as heart and respiratory rate, UAVs should detect victims’ mobility and consciousness from the video data. This paper presents an algorithm combining deep learning with image processing techniques to detect human bodies for further (un)consciousness classification. The algorithm was tested in a 20-subject group in an outside environment with static (RGB and thermal) cameras where participants performed different limb movements in different body positions and angles between the cameras and the bodies’ longitudinal axis. The results verified that the algorithm performed better in RGB. For the most probable case of 0 degrees, RGB data obtained the following results: Mathews correlation coefficient (MMC) of 0.943, F1-score of 0.951, and precision-recall area under curve AUC (PRC) score of 0.968. For the thermal data, the MMC was 0.913, F1-score averaged 0.923, and AUC (PRC) was 0.960. Overall, the algorithm may be promising along with others for a complete contactless triage assessment in disaster events during day and night.

## 1. Introduction

Disaster medicine frequently is characterized by lack of personal resources where an efficient and keen triage is essential for rescuing survivors. During triage, individuals are categorized according to injury severity and available medical resources [1]. In catastrophic situations, medical responders are overwhelmed and rationing healthcare resources is inevitable [1]. The focus is not on individuals but on populations, and these approaches’ standard goal is to provide optimal services for the largest number of people [1,2].

Nowadays, triage is usually performed in three stages [3]: The primary triage is at the scene of the incident where an emergency technician carries it out, then aims the prompt assessment of the injured person, and conducts the rapid transfer to the treatment center. In most mass casualty incidents, triaging systems use tags or colored designations to categorize the victims according to their condition and their treatment need. Secondary triage is used when, due to the large extent of the incident and lack of resources in the pre-hospital, the transmission of the injured person has been prolonged in the scene and triage is performed at the hospital. The third triage is performed by a surgeon or a critical care specialist to prioritize and decide on receiving care services [3].

In the last decades, several technological progresses have been made to optimize triage in these scenarios by: including telemedical services on site with audio-video streaming through a mobile transmission unit [4], smart glasses and augmented reality [5,6], and the development of an artificial intelligence (AI) classification for survival prediction via wearable devices and based on vital signs (while replacing medical judgment indicators) [7]. Other on-ground-based options are rescue robots, rescue workers, and rescue dogs [8]. The first requires invasive equipment and human operators with the possibility of limited access due to restrained mobility [8]. Rescue workers and rescue dogs, in turn, can overcome limited mobility, but need time and quick dislocation to distant locations [8]. As part of the project FALKE, which the German Federal Ministry of Education and Research funded, our research group investigates whether unmanned aerial vehicles (UAVs) equipped with high resolution cameras can be used for a (semi-)automatic triage. Here, a developed triage algorithm proposes a triage tag for each injured person based on their vital parameters’ information obtained remotely with the cameras. Afterwards, a medical professional verifies the recommended triage tag. The speed and altitude of UAVs allied to imaging sensors may be especially adequate for an initial and fast assessment of scenarios with difficult or delayed access (due to physical barriers or Chemical, Biological, Radiological and Nuclear [CBRN] situations) and be suitable for searching for living survivors in the aftermath of a mass casualty incident [8].

In recent decades, different triage algorithms were developed and established to help rescue emergency professionals, avoid deficits in treatment and enable an early assessment at the scene [2,5,9]. Some highly integrated triage systems in the emergency department throughout the world are: Canadian Triage and Acuity Scale (cCTAS), the Australian Triage Score, the Manchester Triage Score, and Emergency Severity Index and Simple Triage and Rapid Treatment (START) [10]. In Germany, the Primary Ranking for Initial Orientation in Rescue Service (PRIOR) algorithm is often used [5]. Here, the patient’s consciousness, breathing, and circulation are qualitatively addressed, which results in an individual triage and non-pathological evaluation [5]. PRIOR triage is divided into three categories as follows: severely injured with immediate treatment priority (category red or I), severely injured with appropriate transport priority (category yellow or II), and easily injured or uninjured (category green or III) [5]. The triage process also may be based on both patient’s vital signs (respiratory rate, oxygen saturation in blood, heart rate, level of consciousness, and body temperature) and their main complaints [11]. Oxygen saturation along with age and level of consciousness were the three variables that best predicted mortality during hospitalization in two studies [11,12,13]. Another study showed that impaired consciousness was a potentially life-threatening emergency and an important factor for patients to enter the emergency department [14]. The Glasgow Coma Scale (CGS) is a scoring system to classify a patient’s conscious state based on three scaled values (eye opening, verbal response, and motor response) [7]. Another triage algorithm Amberg-Schwandorf Algorithm for Primary Triage (ASAV) algorithm takes into account, among other variables, the body posture, consciousness, and if the patient is ambulating [15]. Other methods use the “ability to walk” as a criterion for the categorization, and though it is not a good discriminant, it is suited excellently for performing a rapid but coarse division of patients in tangled scenarios [9]. In fact, patients lacking walking ability must be immediately rescued from the injury zone. For subsequent triage and treatment priority allocation, this discriminate is then unsuitable [9]. In 2021, Álvarez-García and co-workers proposed the concept for an Aerial Remote Triage Algorithm [16]. This includes four steps for victims’ assessment: (1) major bleeding, (2) walking, (3) consciousness, and (4) signs of life (e.g., spontaneous body movements, respiratory movements, coughing, etc.). According to the system, injured people can be classified into four categories: priority 1 (red), priority 2 (yellow), priority 3 (green), and priority * (violet).

Thus, independent of the triage method used, the level of consciousness or unconsciousness is a triage parameter and may be assessed by evaluating the patient’s visual mobility capability or body activity. This work aims to visually detect patient unconsciousness and/or immobility through their mobility capacity and motion detection during daytime and nighttime using visible imaging (RGB) and infrared thermography (IRT, also known as thermal imaging) for each case, respectively. Hence, the current paper presents a new algorithm for contactless detection of human immobility and unconsciousness based on the presumption that if a person is unconscious, they most likely will be motionless. Thus, an algorithm is proposed that combines deep learning with image processing techniques for detecting and tracking human bodies and further consciousness classification. To the best of our knowledge, there is no up-to-date algorithm in the literature for the contactless classification of patient consciousness and/or mobility on disaster survivors.

## 2. Materials and Methods

**Figure 1:**
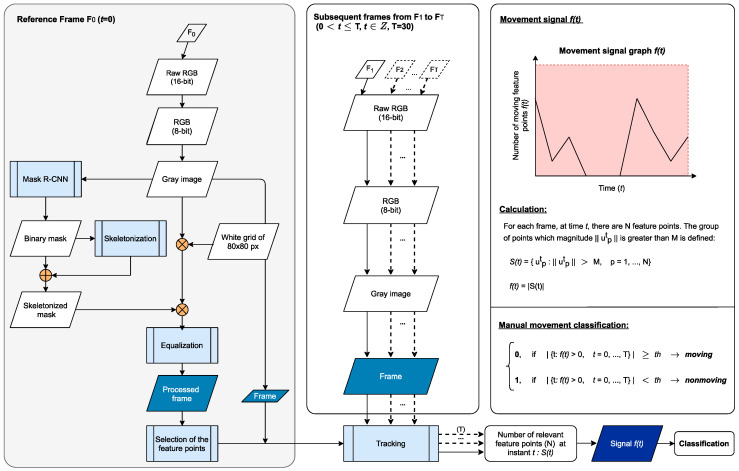
Flowchart of the algorithm steps for the human movement detection in RGB data.

**Figure 2:**
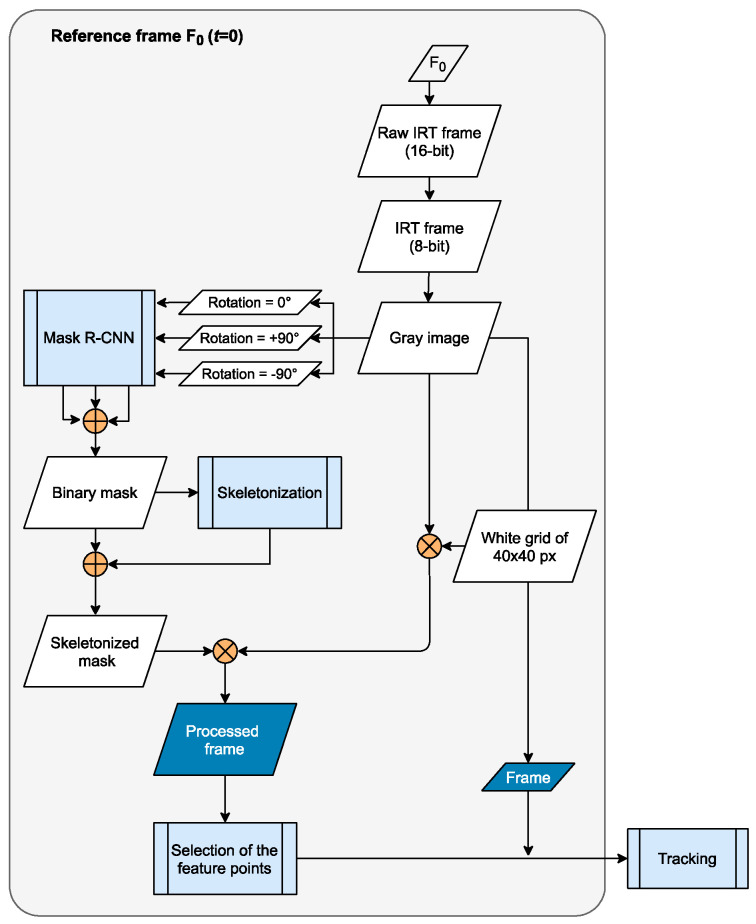
Flowchart of the reference frame’s image processing method for the IRT data. Note there is no equalization process, the white grid applied to the gray image has a size of 40 px, and the different masks obtained from the mask R-CNN are merged (from different rotation angles of the gray image).

This section presents the algorithm developed for consciousness detection. It was optimized for two imaging techniques: RGB and IRT. The current approach is based on tracking the feature points throughout the videos. For both imaging techniques, the process is similar, differing only in the image processing of the reference frame F_0_ in the first step. Here, in the reference frame, the IRT or RGB frame is preprocessed to have the most feature points dispersed in the region of interest (ROI), which is the human body. Forthwith, in a second step, the feature points will be tracked in the subsequent frames from F_1_ to F_T_ where 0<t≤T, t∈ℤ, T=30. All frames are converted in grayscale. Afterwards, the movement signal will be extracted and classified as “moving” or “nonmoving.” The flowchart diagram describing the process for the RGB data is displayed in Figure 1. In Figure 2, only the first step for IRT data is described because it is the only one that differs. 

The difference in imaging processes include for RGB data: the existence of the equalization process applied to the skeletonized mask applied to the gray image, and the dimension of the white grid, which is 80 × 80 px. Relative to the IRT data, there is no equalization applied to the image, the dimension of the white grid is of 40 × 40 px, the input to the mask R-CNN (region-based convolutional neural networks) model has three channels (all gray color), and there is a merge process of all the masks obtained from the gray image in different image rotation levels (0°, +90°, −90°). These variations occur due to different image resolutions, image type, and camera lens between the infrared and visible cameras. Both the grid and skeletonization processes are applied in a further step for a better contrast when selecting the feature points inside the human body region.

The present approach was implemented in Python 3.7 and tested on a 64-Bit Windows 10 computer with an octa-Core Intel^®^ Core™ i5-8600K CPU @ 3.60 GHz, 16 GB RAM with an NVIDIA GTX 1070 (8 GB AMD GDDR5) manufactured by NVIDIA Corporate based in California, United States of America. The analysis of the data was carried out offline.

### 2.1. Preprocessing of the Reference Image and Subsequent Frames

The approach’s first step consisted of converting the recorded frames (BAYER pattern (16-bit)) into RGB format (8-bit) and then into a gray image. Subsequently, the gray frame served as input for the mask R-CNN model. The mask R-CNN by He et al. [17] is a convolutional neural network (CNN) that detects objects in an image and generates a high-quality segmentation mask for each instance. It extends faster R-CNN by adding a branch, which predicts segmentation masks on each region of interest to the existing one for classification and bounding box regression. Faster R-CNN is composed of two stages: (1) region proposal network and (2) fast R-CNN model. The former is a neural network that proposes candidate object bounding boxes in a particular image. The latter extracts features using ROI pooling from each candidate box and performs classification and bounding-box regression. The final step of the mask R-CNN consisted in applying a fully connected network to each ROI to predict a segmentation mask. As pixel-level segmentation requires more fine-grained alignment than bounding boxes, mask R-CNN replaces ROI pooling with an ROI align layer. In the ROI align method, the bilinear interpolation was used to compute the image value on the pixel point whose coordinate is a floating-point number, thus mapping the ROI is more precise. Please note that the mask R-CNN model’s pre-trained weights were based on the COCO dataset (http://cocodataset.org, accessed on 15 September 2021) [18]. This is a large dataset used for target detection and image segmentation, including 328K images and 91 target categories. In sum, mask R-CNN served to not only recognize and classify a human being as a “person” in the images, but also compute its mask. The detection threshold was 70%, which meant that all the proposals with less than 0.7 confidence were ignored. 

The next step of the algorithm consisted of skeletonizing the binary mask (output of the mask R-CNN). There are three major categories of skeletonization techniques: (1) skeleton is extracted based on distance transforms or region in an image, (2) calculating the Voronoi diagram the boundary points generate, and (3) through morphological thinning techniques based on erosion operations [19]. For this aim, image morphological operations were performed by using binary structuring elements (SE), that is, a small matrix or kernel [20]. For instance, erosion is used to shrink objects in an image and remove noise, while dilation adds pixels to the objects’ boundaries. Let *A* be the set of foreground pixels (i.e., objects) and *B* the SE, with *A* and *B* sets in $z^{2}$, the erosion of *A* by *B*, denoted A⊖B, is defined as:

(1) $$A⊖B=\left\{z|{\left(B\right)}_{z}\subseteq A\right\},$$

where the *z*’s are foreground values (ones). In other words, the erosion of *A* by B is the set of all points *z* such that *B*, translated by *z*, is contained in *A* [20]. As for dilating *A* by *B*, denoted as $A⊕B,\;\mathrm{with}\;A\;\mathrm{and}\;B\;\mathrm{as}\;\mathrm{sets}\;\mathrm{in}\;z^{2},\;\mathrm{it}\;\mathrm{is}\;\mathrm{defined}\;\mathrm{as}$:

(2) $$A⊕B=\left\{z|{\left(\hat{B}\right)}_{z}\cap^{\;}A\neq \emptyset \right\}.$$

Dilation is based on reflecting *B* in its origin and translating the reflection by *z* as in erosion. In other words, dilating *A* by *B* is the set of all displacements *z*, such that the foreground elements of $\hat{B}$ overlap at least one element *A*. These morphological operations take part in the skeletonization process according to Lee et al. [21]. First, an erosion followed by dilation known as opening, is performed to the original image. Then, the opening resulting image is subtracted from the original image, resulting in a temporary image. Forthwith, the original image is eroded, and the skeleton is refined by computing the union of the current skeleton and the temporary image. This step creates the skeletonized mask.

To the gray image, a white grid is applied before the multiplication process. Afterwards, to adjust image intensities and enhance contrast, the resulted image *f* from the multiplication of the skeletonized mask was equalized with the grided frame. Please note that no equalization was performed for the IRT frames. Let image *f* be represented as $m_{r}$ by $m_{c}$ matrix of integer pixel intensities (*n*) ranging from 0 to *L −* 1 [20]. Let *p* denote the normalized histogram of *f* with a bin for each possible intensity so that:

(3) $$p_{n}=\frac{number\;of\;pixels\;with\;intensity\;n}{m_{r}\times m_{c}},\;n=0,\;1,\;\ldots ,\;L-1$$

The histogram of the equalized image *g* is defined by:

(4) $$g_{i,f}=floor\;(\left(L-1\right)\overset{f_{i,j}}{\underset{n=0}{\sum }}p_{n}),$$

where *floor ()* rounds down to the nearest integer. This is equivalent to transforming the pixel intensities, *k*, of *f* by the function:

(5) $$T\left(k\right)=floor\;(\left(L-1\right)\overset{K}{\underset{n=0}{\sum }}p_{n}).$$

Thinking of the intensities of *f* and *g* as continuous random variables *X* and *Y* on [0, *L* −1], *Y* is defined by:

(6) $$Y=T\left(X\right)=\left(L-1\right)\int_{0}^{X}p_{X}\left(x\right)dx,$$

where $p_{X}$ is the probability density function (PDF) of a random variable *X*. The function *T* is the cumulative distributive function (CDF) of *X* multiplied by (*L* − 1) [20]. The function *T* is always positive, and it corresponds to the uniform histogram of the resulting equalized image [20]. *Y* corresponds to the image resultant of the equalization process. 

### 2.2. Selection and Tracking of the Feature Points

In the next step, the feature points were selected and then tracked using the Shi-Tomasi corner detector [22]. For a window (*W*) located at (*x,y*) with a pixel intensity of *I (x,y),* the scoring function calculates the score for each pixel to be considered a corner by:

(7) $$f\left(x,y\right)=\sum \left(I(x_{k}+y_{k}\right)-I\;{\left(x_{k}+\Delta x,y_{k}+\Delta y\right)}^{2}\;where\;(x_{k}+y_{k})\;\epsilon \;W.$$

When scanning an image with a window and an area is above a minimum value, a corner is selected at that point through:

(8) $$R=\min\left(\lambda_{1},\lambda_{2}\right).$$

Thus, *R* is the score, where $\lambda_{1}\;\mathrm{and}\;\lambda_{2}$ are eigenvalues of the scoring function. If *R* is greater than a certain predefined value, it can be marked as a corner. This works as follows: if both eigenvalues are greater than a certain value, the pixel is considered a corner; if either $\lambda_{1}\;\mathrm{or}\;\lambda_{2}$ is less than the required minimum, the pixel is considered an edge; or if both eigenvalues are less than the required minimum, the pixel is considered “flat” [23].

The algorithm finds *N* (set to a maximum of 90) strongest corners in the gray image with a distance of at least 10 pixels. After selecting the corners, the aim is to compute their trajectories in consecutive frames. Using the Lucas–Kanade optical flow method [24], the selected feature points were iteratively tracked throughout the video frames. The algorithm identifies the feature points’ location in a specifically sized window around the feature points in the next frame. Displacing the feature points creates feature vectors. Considering the intensity of a pixel *I(x,y,t)* at instant *t* in time, that moves by distance *(dx,dy)* in next frame at *dt* time, and considering that the pixels do not change intensity, it can be stated that:

(9) I(x,y,t)=I(x+dx, y+dy).

Using the Taylors series approximation, removing the common terms and dividing by *dt*, results in the following equation:

(10) $$f_{x}u+f_{y}v+f_{t}=0.$$

Here, $f_{x}$ and $f_{y}$ are image gradients and $f_{t}$ is the gradient along time. To obtain the vector *(u,v)* values, the least square fit method is used, resulting in the equation:

(11) $$\left[\begin{array}{c} u\\ v \end{array}\right]={\left[\begin{array}{cc} \underset{i}{\sum }f_{x_{i}}{}^{2} & \underset{i}{\sum }f_{x_{i}}f_{y_{i}}\\ \underset{i}{\sum }f_{x_{i}}f_{y_{i}} & \underset{i}{\sum }f_{y_{i}}{}^{2} \end{array}\right]}^{-1}\left[\begin{array}{c} -\underset{i}{\sum }f_{x_{i}}f_{t_{i}}\\ \underset{i}{\sum }f_{y_{i}}f_{t_{i}} \end{array}\right],\;$$

where *u* and *v* are vector values in *x* and *y*, respectively. Let vector *u* be derived through the gradient in *x* and *y* coordinates, the image velocity at *x* is also known as optical flow at *x* [25].

### 2.3. Extracting the Movement Signal and Classification

The movement signal was obtained through all the read frames along the recorded videos of period *T =* 30 s. First, for each frame, the algorithm computed the feature vectors’ vertical and horizontal gradients. The gradient’s magnitude was also calculated for each feature vector. Let the two-dimensional vector be *u =* ($u_{1},u_{2}$) and its magnitude be ||u||. The tracking points (*N*) which magnitude at instant *t* $\left||u_{p}^{t}|\right|$ was higher than a certain magnitude *M* (distance/dt) in between the frames and was selected resulting in *S(t)*. The latter is the group of feature points in which magnitude is higher than *M*. The value of *M* was set to 100 for visible data and 450 for infrared data. The *S(t)* function at instant *t* is defined by:

(12) $$S\left(t\right)=\left\{u_{p}^{t}:\;||u_{p}^{t}||>M,\;p=1,\ldots ,\;N\right\},\;(0<t\leq T,\;t\in \mathbb{Z}).$$

Then, the signal *f* along the period of 30 s was the counting of each instant’s relevant feature points, or the number of the selected feature points in the group *S(t)* for each instant:

(13) f(t)=|S(t)| 

Finally, the number of peaks of the signal during the video duration of 30 s (*N* = 30) was counted and was only considered if it was lower than the threshold *th* (set to 3). Therefore, if the number of peaks was lower than *th*, the algorithm interpreted the signal as a nonmovement and stated that the person was “nonmoving”. Otherwise, it considered as “moving.” The “nonmoving” or potentially unconscious person was labeled as 1 and “nonmoving” or conscious person was labeled as 0:

(14) $$Classification=\{\begin{array}{l} 0,\;\;if\;\left|\left\{t:f\left(t\right)>0,\;\;t=0,\ldots ,T\;\right\}\right|\;\;\geq \;3\;\;\to moving\\ 1,\;\;if\;\left|\left\{t:f\left(t\right)>0,\;\;t=0,\ldots ,T\;\right\}\right|\;\;<\;3\;\;\to nonmoving \end{array}$$

where  (0<t<T, t∈ℤ). At the end, the algorithm returned a value as “moving” or “nonmoving,” which was considered either as conscious or potentially unconscious, respectively. For a clearer and more practical follow-up and understanding, the labels “moving” and “movement” were used for mobility/consciousness detection (negative prediction) and “nonmoving” and “no movement” for immobility/unconsciousness detection (positive prediction). This labeling is due to how unconsciousness detection is our aim, and thus, a positive prediction.

**Figure 3:**
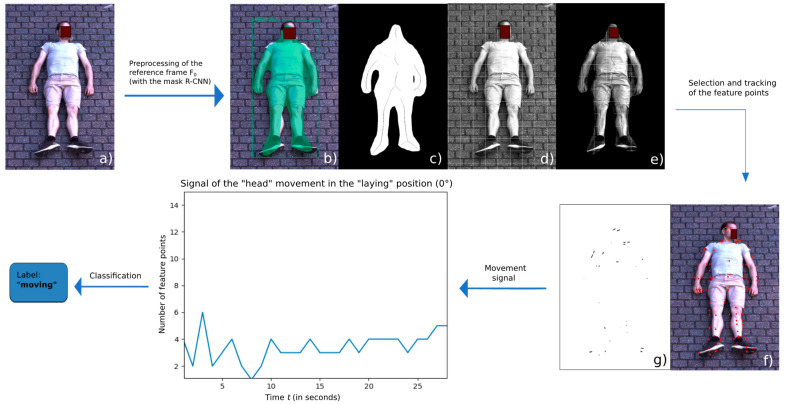
Intermediary RGB images throughout the algorithmic process: (**a**) the original RGB frame, (**b**) the mask and bounding box given from mask R-CNN, (**c**) the binary skeletonized mask, (**d**) the white grid on the gray image, (**e**) the processed image prepared for the selection of feature points, (**f**) the selection of feature points, and (**g**) the movement lines in between two frames of the feature points (essentially “head” movement). The obtained movement signal through time is displayed in the graphic, and, in this case, the person is classified as “moving”.

All steps to the final classification of the human movement as negative or positive in the RGB are displayed in Figure 3. First, from the mask obtained from the mask R-CNN, its skeleton and a white grid were added to the same mask, which was then applied to the gray image. This first step was preprocessing the videos’ first frame. Afterwards, the feature points from the frame 0 were selected and then tracked in the gray subsequent frames. Finally, processing the frame data resulted in a movement signal along the 30 s recording time and later in classifying a moving/nonmoving person.

### 2.4. Statistical Methods

For the human movement detection, a binary classification using the labels movement/consciousness and no movement/unconsciousness were used. For each of the different movements the patients performed, the positive label and the negative label corresponded to “no movement” and “movement,” respectively. Thus, the ground truth of the data was no movement when there was no movement, and movement for each of the limbs’ movement performed (head, one arm, both arms, one leg, both legs, and slight movement). As one may note, the dataset was imbalanced because the ratio of positives to negatives was 1:6. For a clearer understanding and more appropriate analysis of the algorithm’s performance, various statistical parameters were investigated and the following were chosen: F1-score, AUC (PRC), MCC, Cohen’s kappa or kappa, and balance error rate (BER).

Let “TN,” “TP,” “FN,” and “FP” represent “true negative,” “true positive,” “false negative,” and “false positive,” respectively. The total number of samples in the classification is N (=TN + TP + FN + FP). In such an imbalanced dataset, where the number of negative samples is large, the false positive rate increases slower. Typical calculated metrics include precision, recall, and accuracy. However, these alone are not a good measure when evaluating an imbalanced dataset, thus leading to an imbalanced precision and recall. Therefore, for a comprehensive evaluation of the classification model metrics, the harmonic mean of precision and recall ($F_{\beta }$-score) was calculated by [26]:

(15) $$CF_{\beta }=\left(1+\beta^{2}\right)\;\cdot \;\frac{precision\;\cdot \;recall}{\left(\beta^{2}\cdot \;precision\right)+recall}.$$

The beta parameter allowed us to control the tradeoff of importance between precision and recall. *β* < 1 focuses more on precision, while *β* > 1 focuses more on recall metrics [26]. Regarding the F1-score, *β* equals 1, and both recall and precision have the same weight on evaluating the process.

Another statistical metric evaluated was Cohen’s kappa *(k)*, which is another popular consensus metric of interrater reliability that estimates the degree of consensus between two judges and determinates whether the agreement level exceeds what would be expected to be observed by chance alone [27,28]. A kappa value of 0 does not indicate that the two judges did not agree at all but rather that the two judges did not agree with each other any more than would have been predicted by chance alone. Again, the kappa coefficient is a highly useful statistic when most observations fall into a single category. Supposing the correct recognition rates (*CR*_1_ and *CR*_2_) and error rates (*E*_1_ and *E*_2_) from a confusion matrix are defined by

(16) $$\begin{array}{c} CR_{1}=\frac{TN}{N},\;\;\;\;CR_{2}=\frac{TP}{N},\\ E_{1}=\frac{FP}{N},\;\;\;\;E_{2}=\frac{FN}{N}, \end{array}$$

and supposing the relationship of the population rates *p*_1_ and _p2_ is

(17) $$p_{1}=CR_{1}+E_{1},\;\mathrm{and}\;p_{2}=CR_{2}+E_{2},\;$$

Cohen’s kappa is described as follows and within the range [−1, 1]:

(18) k=(Pa−Pc)/(1−Pc), where

(19) $$P_{a}=\frac{TN+TP}{N}\;and$$

(20) $$P_{c}=p_{1}\frac{TN+FN}{N}+p_{2}\frac{TP+FP}{N}.$$

Kappa or *k* is a metric used to measure the strength of inter-rater agreement, for which the maximum agreement is 1 and 0 represents a random agreement [29,30]. 

The Matthews correlation coefficient (MCC) is a measure that is not influenced by imbalance test sets since it considers accuracies and error rates mutually on both classes and involves all values of the confusion matrix. The MCC ranges from 1 for a perfect prediction to −1 for the worst possible prediction. A value of 0 indicates a model that performs randomly [31]. Some authors consider this metric as the best singular assessment metric and especially suitable to the case of imbalanced data [32]. It is defined by:

(21) $$\mathrm{MCC}=\frac{TP\cdot TN-FP\cdot FN}{\sqrt{p_{1}p_{2}N^{2}\left(TN+FN\right)\left(TP+FP\right)}}.$$

The balance error rate (BER) is measured rather than the often used error rate since previous studies have shown that the error rate does not change in datasets with unequal numbered classes [33]. The BER is considered a “proper” measure for processing class-imbalanced problems [31]. It is defined as it follows:

(22) $$\mathrm{BER}=\frac{1}{2}\left(\frac{E1}{p_{1}}+\frac{E2}{p_{2}}\right).$$

In a study carried by [34] showed that accuracy, F1-score, kappa, and ROC curve were are skewed distributions in imbalanced data [34]. Cohen’s kappa was affected by skew in either direction, while F1-score was affected in only one direction.

Finally, commonly used measures of classifier performance for visual graphical data are accuracy, error rate, and area under the receiver operating characteristics (ROC) curve (AUC). However, recent studies [33,35,36] have shown that ROC can be misleading when this last applied to imbalance scenarios, due to its overly optimistic performance. In fact, ROC plots can be deceptive with respect to conclusions about the reliability of classification performance, due an intuitive but wrong interpretation of specificity in imbalanced datasets [32,33,36]. Studies have shown that only precision, PRC, MMC, and F-beta scores vary between datasets, while most other measures stay unchanged (ROC, concentrated ROC [CROC], cost curves [CCs], error rate, accuracy, FPR, and accuracy) [32,33,36]. As compared to the traditional ROC, PRC changes not only with the ratio of positive and negatives in the dataset but also with the AUC score of the PRC as well. The latter is denoted as AUC (PRC) and will be evaluated in this study [33,34,37].

### 2.5. Experimental Protocol 

**Figure 4:**
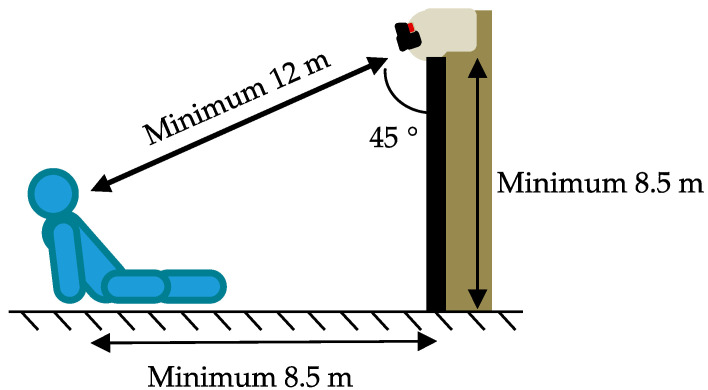
Sketch of the experimental setup on the floor (using static cameras).

An experiment was carried out in an outdoor uncontrolled environment with static infrared and visible cameras: a FLIR BOSON 640 (640 × 512 px image resolution with spectral range 7.5–14 µm) and Allied Vision Mako G-234C (1080 × 1920 px image resolution) with a frame rate of 30 and 15 fps, respectively. These were sat atop a tripod and positioned so that the angle between them and the floor was 45°, and the diagonal distance to the patients was approximately 12 m, as depicted in Figure 4. Here, it was important to have a full human body in the field of view of the cameras. The setup was dimensioned so that it could be as similar as possible to the setup using a UAV with cameras hovering over the wounded people. 

The tripod was placed on the second floor of the Acute Care Innovation Hub Institute to replicate the camera’s angle and height toward the floor when using an UAV. In this cast, 20 healthy volunteers (six women and 14 men) between the ages of 22 and 36 (27.40 years ± 3.84) participated in the current experiment. Each participant’s temperature was checked (using an infrared FLIR E95 camera), and basic individual and weather parameters were collected. The subject group had an average height of 180.40 cm ± 9.55, average weight of 78.3 kg ± 15.02, and average body temperature of 36.69 °C ± 1.28. The average weather conditions were 26.50 °C ± 4.34 temperature, 31.15% ± 4.21 relative humidity, and 22 km/h ± 6.94 wind velocity. 

**Figure 5:**
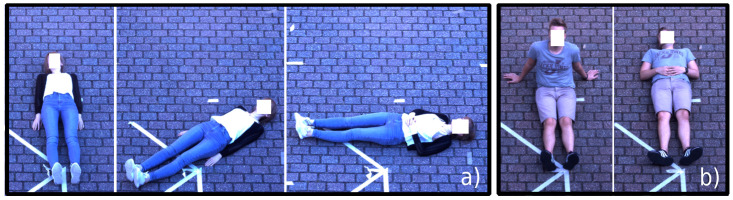
(**a**) Laying positions seen from different angles between the longitudinal axis of the human body and the cameras (0°, 45°, and 90° clockwise); (**b**) sitting and laying positions in a 0° body sagittal plan rotation toward the camera.

The same protocol was performed using three body angles toward the camera (clockwise rotation on the longitudinal axis of the human body at 0°, 45°, and 90°, as depicted in Figure 5a). For each angle, the subjects posed in two positions: “sitting” and “laying” (as displayed in Figure 5b). For each position, a set of seven movements was performed (“no movement,” “head,” “one arm,” “both arms,” “one leg,” “both legs,” and “slight” movement) for 30 s each.

## 3. Results

Most of the videos were recorded according to the study protocol. However, due to technical issues in the image calibration of the thermal cameras, only IRT videos from 12 probands had sufficient quality to be evaluated. 

All of the human subjects performed seven movements in two positions (sitting and laying) for each angle (0°, 45° and 90°) for a 30 s duration. Thus, 42 video recordings were made per subject. Overall, for the group of 20 individuals, there were 280 video samples for each angle, of which 40 videos labeled as “no movement” and the remaining 240 categorized as “movement.” In sum, 840 total RGB videos were collected. The statistical metrics from the RGB data are presented in Table 1.

The ratios of correctly predicted classes for each position, angle, and movement are displayed in Table 2. The ratio values of correctly predicted classes is 1 when there is the greatest and perfect correspondence between the actual and predicted value, and 0 for correspondence at all. Notably, the algorithm performed worse for the “slight” movement, especially in the “laying” position, where it predicted “nonmoving” instead of “moving.” As for the “no movement” pose, it failed for the “sitting” positions at 0° and 45°. Additionally, the algorithm failed once for the “one arm” movement detection. 

All the positive cases were correctly predicted at both 0° and 45°, while the number of FPs was similar for all the angles. Recall values was 1.0 and higher at 0° and 45°, while precision was higher for 0° than it was for the other two angles. Accuracy values ranged between 0.958 and 0.976, with 0° having the greatest value. The F1-score ranged from 0.889 to 0.923 and was the highest at 0°. AUC (ROC) scores were similar, ranging from 0.936 to 0.960, and was the highest at 0° as well. Finally, kappa and MMC scores had higher variance, between 0.938 and 0.909 as well as 0.840 and 0.913, respectively. The BER was the lowest for 0°, followed by 45° and then 90°; it ranged between 0.014 and 0.059.

Overall, the algorithm had better statistical metrics at 0° and 90°. The number of FNs was also lower for these two angles, while the number of FPs was greater for 0° and 45°. The F1-score was higher for 0° and 90°, ranging from 0.914 to 0.988, and the AUC (PRC) varied from 0.965 to 0.994. The kappa and MMC scores presented equal values at each angle, varying from 0.899 to 0.986. The BER values were between 0.002 and 0.046. At 0° and 90°, the F1-score, kappa, and MMC scores were higher and the BER was lower. 

Regarding the IRT results of the data available from 12 individuals, 168 videos samples were collected from each angle, with 24 corresponding to the “no movement” class and the remaining 144 to the “movement” class. Table 3 shows the statistical metrics for the infrared data.

Table 4 displays the ratios of correctly predicted classes for each position, angle, and movement. The incorrectly predicted classes mainly involved the “slight” movement, with a small tendency toward the “sitting” position. For the “no movement” pose, the algorithm failed only for 0° at the “sitting” position. In one video, the classifier categorized the “one arm” movement as “nonmoving” instead of “moving”.

For comparison purposes, the statistical metrics for the RGB data were calculated using only the subjects for whom infrared videos were available. These results are displayed in Table 5. Overall, similar results were obtained to those presented in Table 5. The F1-scores ranged from 0.980 to 0.917 and were the highest at 90°. AUC (PRC) had similar scores for all angles, from 0.982 to 0.988. Kappa and MMC ranged from 0.903–0.976 and 0.903–0.976, respectively. Lastly, BER was lowest at 90°, followed by 0° and 45°, ranging between 0.003 and 0.049.

Table 6 displays the ratios of correctly predicted classes for each position, angle, and movement. Here, the wrongly predicted labels involved the “slight” movement in the “laying” position, for which the algorithm predicted “nonmoving” instead of “moving.” Additionally, two “no movement” videos were wrongly predicted as “moving” videos at 45°. There were no incorrectly predicted videos for the “moving” videos except for “slight” movement.

## 4. Discussion

In general, the presented algorithm obtained good results with both the IRT and RGB data for detecting immobility on people using upper static cameras. 

In the RGB 20-subject data results, the mask R-CNN detected and segmented all the subjects with very good accuracy. However, in some videos, the mask R-CNN could not segment the bodily extremities (hands and feet) with great precision. From the 12 wrongly predictions, 4 were FNs and 8 were FPs. The four videos wrongly predicted as “moving” instead of “nonmoving” (FN) were due to some clothing movement caused by the wind or to sudden sunlight changes. The very intense bright light led to a greater number of homogeneous pixels, consequently hindering the tracking of the feature points. The FNs were mainly in the “sitting” position; and, the algorithm performed worst at 45°. In turn, the FPs were mainly in the “laying” position, and no major differences were observed between all three angles. Additionally, the majority of the FPs were among the “slight” movement videos, where the subjects performed “slight movements,” such as moving their hand, fingers, or feet. These videos were expected to have fewer moving feature points and with less magnitude, making these movements more prone to being predicted incorrectly. The main reasons for the FPs and FNs were non-detection of fully body extremities by the mask R-CNN, insufficient magnitude of the feature points for them to be considered relevant moving ones, and an insufficient number of feature points. There were more FPs than FNs. In fact, having more FPs is preferable. It is more important to minimize FNs or the cases where unconscious people are classified as conscious, for a greater impact on their survival in a disaster scenario.

Overall, the developed algorithm predicted “movement” and “non-movement” with high kappa, MMC, and F1-score values and low BER. The kappa and MMC values were lower than the F1-scores were. The statistical metrics show that the MMC values are symmetrical (they consider accuracies and error rates mutually on both classes) and involve all values of the confusion matrix. The MMC values correspond to the correlation between both true and predicted values and translates the probability of having a perfect prediction, ranging from [−1 to 1]. Data evaluation showed equal MMC and Kappa values of 0.943, 0.899 and 0.986 for 0°, 45°, and 90°, respectively. According to the interpretation of the metric’s creators Landis and Koch [29], Kappa values are considered almost perfect as values belong to interval [0.81–1.00]. AUC (PRC) values were also good for all three angles, ranging from 0.965 to 0.994. Thus, the algorithm appears to be promising for detecting immobility in people and potential unconsciousness.

Regarding the IRT data (including only videos from 12-subjects), the mask R-CNN computed and segmented human bodies with less precision. By comparison, for the RGB data, body extremities were less segmented, and full limbs sometimes were not segmented. This might be due to the training of the neural model with RGB images instead of IRT images. Overall, the algorithm developed for thermal images had good performance but was less accurate compared to the results obtained for the RGB data. Among the 504 videos, there were 17 wrongly predicted values, 2 FNs (both at 90°) and 15 FPs (mostly for “slight” movement at the three angles). The FPs were in the “sitting” and “laying” positions, while the FPs tended to be in the “sitting” position. The algorithm performed the worst at 90° and the best at 0°. The results from 45° were better relatively to those of 90° by presenting no FNs. The algorithm mainly failed for the following reasons for the IRT data and for FPs. The mask failed to detect the bodily extremities as part of the human body, so the number of moving feature points was insufficient for the signal to be considered motion for the recorded time period. The remaining FNs occurred when the movement was so slight that the magnitude of the feature points was insufficient for the points to be considered moving feature points. Both incorrect predictions might be due to the grayscale from the infrared information (in comparison to the RGB scale from visible frames) and the feature points consequently being harder to track, lower image resolution/contrast, and the fact that the mask R-CNN was pre-trained with RGB data and consequently less suitable for infrared human detection. Regarding the metrics, videos recorded in 0° had the best kappa (0.909), MMC (0.913), F1-score (0.923), and BER (0.014) results. For the other angles, the kappa scores were between 0.838 and 0.868, MMC was between 0.840 and 0.876, F1-scores were between 0.863 and 0.889, and BER was between 0.021 and 0.059. Most of the incorrectly predicted values were FPs, which again has less impact in real scenarios. Although the algorithm performed worse with IRT videos during the daylight experiment, the advantage of using infrared thermography, rather than visible imaging, is the possibility of utilizing it at nighttime or scenarios with limited visibility or natural light, such as dusty and foggy conditions.

An evaluation using the RGB data from 12 subjects was performed to compare both imaging technologies. Globally, the algorithm performed better on the RGB data, with MMC scores of 0.943, 0.899, and 0.986 at 0°, 45°, and 90°, respectively. However, the MMC score obtained from the IRT videos was sufficiently good, with 0.913, 0.876, and 0.840 at 0, 45°, and 90°, respectively. The number of FPs and FNs was greater than 10 for the IRT data group. This can be due to the lower contrast among the IRT images, which can lead to less accurate segmentation, less prominent edges in the images, and a lower number of selected feature points, which compromise the tracking performance and classification process. In general, the algorithm performed worse at detecting and recognizing “slight” movement as movement. Most of the FNs were in the “sitting” position, while most of the FPs were in the “laying” position.

For further algorithm development, the mask R-CNN may be refined and be trained with IRT data, or another model pre-trained on infrared images can be applied. The magnitude threshold for feature points to be considered moving ones could be investigated. Additionally, different environments and ground temperatures may be studied, which may influence mask segmentation and classification in IRT images using mask R-CNN. In addition, to the best of our knowledge, there is not up to now any open dataset which meets our requirements relatively to acquisition condition (distance and angle of the cameras toward participants). Thus, to guarantee a more reliable analysis of the algorithm’s performance, more subjects should be enrolled in future studies. The algorithm was tested using higher-level static cameras to validate the algorithm. Testing is planned using cameras placed in a hovering drone over simulated injured people in a set-up catastrophe scenario. In this case, some future main obstacles include the drone’s movement and camera calibration toward the image background as well as how the wind provoked by the drone influences the movement of the probands’ clothes. Other interesting experimental setups would include testing on partially occluded human bodies, testing in different laying positions (supine, prone, or side-lying), or identifying moving limbs. This could lead to more precise information being delivered to rescuers. 

Lastly, to the best of our knowledge, there is no other study or method in literature to assess consciousness detection based on human motion and remote imaging in both infrared and visible imaging, and also specifically when using an UAV. Therefore, this work can be used as preliminary study and a staircase for future algorithm development to be applied in remote and efficient triage algorithms in catastrophic medicine, as well as in other medical fields. In fact, it can provide information about one common triage parameters, i.e., consciousness or/and mobility when categorizing injured people. 

## 5. Conclusions

In this paper, an algorithm was proposed that proved capable of detecting human body immobility through visible and infrared technologies in an outside environment using static cameras. This could be useful for detecting unconsciousness in a disaster scenario. The system is a combination of image-processing techniques along with deep learning. It assesses, for two image modalities, one of the four main parameters (major bleeding, walking, consciousness, and signs of life) essential for triage, according to Álvarez-García et al. [16]. It could be used during daytime and nighttime with RGB and IRT cameras.

In general, the classifier performed better for RGB imaging but still performed well for thermal imaging. It had high MMC and kappa scores for both imaging technologies, especially for the most expected case of a 0° camera angle toward the human body, when a drone evaluates each person during a rescue. Clearly, the algorithm detected more FPs than FNs, which is preferable because classifying wrongly moving people has less of an impact on rescuing people than wrongly identifying injured people who are not moving would. It mainly failed when the participants performed “slight” random movements. As for the FNs, most of them were in the “sitting” position for FNs and in the “laying” position for FPs. For the most expected angle between each human body and the camera (0°), the algorithm had high MMC values of 0.943 and 0.913 with RGB and IR imaging, respectively.

In conclusion, this preliminary study evaluated the performance of the proposed algorithm, using two image modalities, to detect unconscious victims in an outside environment using static cameras. In a future study, the algorithms should be tested using video data acquired from a UAV. The purpose is to use RGB imaging for daytime rescues and IR imaging for nighttime rescues. Indeed, graphic techniques as in the form of smart photographs have still a lot to offer, especially in disaster medicine, either by using solely or merging both AI and image processing techniques. Some existing detectors in literature are already being applied in the health monitoring field including heart rate, respiratory rate, and eye blink detection algorithms. With the presented algorithm, we aim to evaluate automatically and objectively (instead of subjectively) one common triage parameter (consciousness) for the purpose of triage and rescue optimization, while easing the work of paramedics. In this wise, this algorithm along other vital parameters detectors, could provide a quicker and more reliable information to rescuers and efficiently prioritize the most severely ill patients. Specifically, using an UAV, such system could remotely suggest an holistic patient categorization and well-being report before the medical team arrives the disaster site. Lastly, remote imaging systems can be applied to areas of expertise other than the medical field.

## Figures and Tables

**Table 1:** Statistical metrics for the RGB data from the whole study group for three angles between the longitudinal axis of the body and the cameras.

Metrics	Angles		
	0°	45°	90°
Confusion matrix $\left(\begin{array}{cc} TN & FP\\ FN & TP \end{array}\right)$	$\left(\begin{array}{cc} 237 & 3\\ 1 & 39 \end{array}\right)$	$\left(\begin{array}{cc} 236 & 4\\ 3 & 37 \end{array}\right)$	$\left(\begin{array}{cc} 239 & 1\\ 0 & 40 \end{array}\right)$
F1-score	0.951	0.914	0.988
AUC (PRC)	0.968	0.965	0.994
kappa	0.943	0.899	0.986
MMC	0.943	0.899	0.986
BER	0.019	0.046	0.002

*TN*: true negatives, *FP*: false positives, *FN*: false negatives, *TP*: true positives.

**Table 2:** Ratio of correctly predicted classes for each position, angle, and movement for the RGB data from the 20-subject group (each 0.05 in the value accounts for a corrected predicted video).

	No Movement	Head	One Arm	Both Arms	One Leg	Both Legs	Slight
Sitting 0°	0.95	1.00	1.00	1.00	1.00	1.00	1.00
Laying 0°	1.00	1.00	1.00	1.00	1.00	1.00	0.85
Sitting 45°	0.85	1.00	1.00	1.00	1.00	1.00	1.00
Laying 45°	1.00	1.00	0.95	1.00	1.00	1.00	0.85
Sitting 90°	1.00	1.00	1.00	1.00	1.00	1.00	1.00
Laying 90°	1.00	1.00	1.00	1.00	1.00	1.00	0.95

**Table 3:** Statistical results for the IRT data of the group of 12 participants for three angles between the longitudinal axis of the body and the cameras.

Metrics	Angles		
	0°	45°	90°
Confusion matrix $\left(\begin{array}{cc} TN & FP\\ FN & TP \end{array}\right)$	$\left(\begin{array}{cc} 140 & 4\\ 0 & 24 \end{array}\right)$	$\left(\begin{array}{cc} 138 & 6\\ 0 & 24 \end{array}\right)$	$\left(\begin{array}{cc} 139 & 5\\ 2 & 22 \end{array}\right)$
F1-score	0.923	0.889	0.863
AUC (PRC)	0.960	0.945	0.936
kappa	0.909	0.868	0.838
MMC	0.913	0.876	0.840
BER	0.014	0.021	0.059

*TN*: true negatives, *FP*: false positives, *FN*: false negatives, *TP*: true positives.

**Table 4:** Ratios of correctly predicted classes for each position, angle, and movement for the IRT data from the 12-subject group (approx. each 0.08 accounts for a correctly predicted video).

	No Movement	Head	One Arm	Both Arms	One Leg	Both Legs	Slight
Sitting 0°	1.00	1.00	1.00	1.00	1.00	1.00	0.75
Laying 0°	1.00	1.00	1.00	1.00	1.00	1.00	0.92
Sitting 45°	1.00	1.00	1.00	1.00	1.00	1.00	0.67
Laying 45°	1.00	1.00	1.00	1.00	1.00	1.00	0.83
Sitting 90°	0.92	1.00	1.00	1.00	1.00	1.00	0.83
Laying 90°	0.92	1.00	0.92	1.00	1.00	1.00	0.83

**Table 5:** Statistical results from the RGB data of the 12-participant group for three angles between the longitudinal axis of the body and the cameras.

Metrics	Angles		
	0°	45°	90°
Confusion matrix $\left(\begin{array}{cc} TN & FP\\ FN & TP \end{array}\right)$	$\left(\begin{array}{cc} 142 & 2\\ 0 & 24 \end{array}\right)$	$\left(\begin{array}{cc} 142 & 2\\ 2 & 22 \end{array}\right)$	$\left(\begin{array}{cc} 143 & 1\\ 0 & 24 \end{array}\right)$
F1-score	0.96	0.917	0.98
AUC (PRC)	0.985	0.982	0.988
kappa	0.953	0.903	0.976
MMC	0.954	0.903	0.976
BER	0.007	0.049	0.003

*TN*: true negatives, *FP*: false positives, *FN*: false negatives, *TP*: true positives.

**Table 6:** Ratio of correctly predicted classes for each position, angle, and movement for the RGB data from the 12-subject group (each approx. 0.08 accounts for a corrected predicted video).

	No Movement	Head	One Arm	Both Arms	One Leg	Both Legs	Slight
Sitting 0°	1.00	1.00	1.00	1.00	1.00	1.00	1.00
Laying 0°	1.00	1.00	1.00	1.00	1.00	1.00	0.83
Sitting 45°	0.83	1.00	1.00	1.00	1.00	1.00	1.00
Laying 45°	1.00	1.00	1.00	1.00	1.00	1.00	0.83
Sitting 90°	1.00	1.00	1.00	1.00	1.00	1.00	1.00
Laying 90°	1.00	1.00	1.00	1.00	1.00	1.00	0.92
